# Chlorhexidine for the Treatment of *Fusarium* Keratitis: A Case Series and Mini Review

**DOI:** 10.3390/jof7040255

**Published:** 2021-03-29

**Authors:** Claudy Oliveira dos Santos, Nicolien M. Hanemaaijer, Jelina Ye, Henrich A. L. van der Lee, Paul E. Verweij, Cathrien A. Eggink

**Affiliations:** 1 Centre for Expertise in Mycology, Department of Medical Microbiology, Radboud University Medical Center, 6525 GA Nijmegen, The Netherlands; n.hanemaaijer@izore.nl (N.M.H.); henrich.vanderlee@radboudumc.nl (H.A.L.v.d.L.); Paul.Verweij@radboudumc.nl (P.E.V.); 2 University Medical Center, Department of Medical Microbiology, University of Groningen, 9713 GZ Groningen, The Netherlands; 3 Department of Ophthalmology, Radboud University Medical Center, 6525 GA Nijmegen, The Netherlands; JYe@rijnstate.nl (J.Y.); cathrien.eggink@radboudumc.nl (C.A.E.)

**Keywords:** *Fusarium*, chlorhexidine, fungal keratitis, contact lenses, treatment

## Abstract

Fungal keratitis is difficult to treat, especially *Fusarium* keratitis. In vitro studies show that chlorhexidine could be an interesting option as monotherapy. We describe a case series of four patients (four eyes) with *Fusarium* keratitis at Radboud University Medical Center (Nijmegen, the Netherlands). The patients were treated with chlorhexidine 0.02% eye drops. The in vitro activity of eight antifungals and chlorhexidine was determined according to the European Committee on Antimicrobial Susceptibility Testing (EUCAST) broth microdilution method. We also reviewed the literature on the use of chlorhexidine in the treatment of fungal keratitis. Topical chlorhexidine was well tolerated, and all patients showed complete resolution of the keratitis upon treatment with chlorhexidine. A PubMed search of the available literature was conducted (last search 8 March 2020) and yielded two randomized clinical trials (natamycin versus chlorhexidine) and one case report addressing the treatment of fungal keratitis with chlorhexidine. Chlorhexidine was found to be safe with regard to toxicity and to be superior to natamycin in the clinical trials. Chlorhexidine showed in vitro fungicidal activity against *Fusarium* and clinical effectiveness in our cases, supporting further clinical evaluation. Advantages of chlorhexidine are its topical application, its general availability, its low costs, its broad-spectrum activity, and its fungicidal mechanism of action at low concentrations.

## 1. Introduction

Fungal keratitis is rarely observed in temperate climates but is a common eye infection in tropical and subtropical areas of the world. Although many fungi have been reported to cause fungal keratitis, *Fusarium* species are the most frequent cause. *Fusarium* species are fast-growing hyalohyphomycetes and are ubiquitous organisms that are present in soil, water, and plants. The most common route of infection is through (micro) trauma or disruptive ocular surface disease. In the temperate climates, we see a rise of *Fusarium* keratitis associated with contact lens wear [1,2,3,4]. Clinical diagnosis and management of fungal keratitis is difficult. Early therapy of localized corneal disease is important to prevent progression to a more aggressive or disseminated infection, which ultimately may lead to permanently diminished visual acuity or monocular blindness. Currently, there is a European guideline for the treatment of invasive fusariosis [5], but unfortunately there is no guideline that addresses therapeutic strategies in fungal or *Fusarium* keratitis.

A case of keratitis due to a probable co-infection of *Fusarium solani* and *Acanthamoeba* led us to investigate whether chlorhexidine (CHX) could be used as a therapeutic agent for fungal keratitis. We performed a range of susceptibility tests and decided to use chlorhexidine as an initial therapy for cases with small superficial infiltrates that were suspected to be of a fungal cause awaiting the outcome of culture. Here, we describe four *Fusarium* keratitis cases in which monotherapy with CHX led to a successful outcome without the need of adding antifungal agents. We report the CHX susceptibility patterns of the isolates and review the literature with regard to the use of CHX in the treatment of fungal keratitis.

## 2. Materials and Methods

We describe a case series of the tertiary care facility Radboud University Medical Center (Nijmegen, The Netherlands) between October 2014 and November 2015. The aim of this series was to assess the clinical effectiveness of CHX in the treatment of patients with fungal keratitis. Samples and clinical data from the patients were collected and processed in agreement with the Declaration of Helsinki.

We included all consecutive patients with fungal keratitis. We considered treatment failure as the need to perform a corneal transplantation or evisceration/enucleation of the affected eye. 

The patients were treated with CHX 0.02% eye drops (Pharmaline, Oldenzaal, The Netherlands), at least with hourly drops and after 3 days 8 times a day, and 2 times during the night for a minimum of 4 weeks. The concentration of CHX was based on the Dutch guideline for the treatment of *Acanthamoeba*, the expert opinion of the ophthalmologist and the in vitro susceptibility data routinely determined by the clinical microbiologist; during the studied time period these data were not yet published. The treatment was discontinued upon complete healing. The main outcome measure for each patient was complete healing of the corneal ulcer. 

The in vitro activity of itraconazole (ITC, Janssen Pharmaceutica, Breda, The Netherlands), voriconazole (VCZ, Pfizer, Brussels, Belgium), posaconazole (POS, Merck & Co, Kenilworth, NJ, USA), isavuconazole (ISA, Astellas Pharma, Northbrook, IL, USA), caspofungin (CAS, Merck & Co, Kenilworth, NJ, USA), micafungin (MCF, Astellas Pharma, Northbrook, IL, USA), anidulafungin (ANI, Pfizer, Brussels, Belgium), amphotericin B (AMB, Bristol Myers Squibb, Utrecht, The Netherlands) and chlorhexidine (CHX, Pharmaline, Oldenzaal, The Netherlands) was determined according to the European Committee on Antimicrobial Susceptibility Testing (EUCAST) broth microdilution method [6]. The minimal inhibiting concentrations (MICs) were determined for amphotericin B, the azoles and chlorhexidine. The endpoint for the echinocandins was the minimal effective concentration (MEC). For CHX, the MIC endpoint was chosen at 100% inhibition, and the minimum fungicidal concentration (MFC) was determined by sub culturing each well that showed complete inhibition of fungal growth in comparison with the growth control well. The MFCs were defined as the lowest concentration of CHX that resulted in ≥99% growth inhibition. 

A PubMed search of the available literature was conducted (last search 8 March 2020). Publications were included if CHX was used as treatment of fungal keratitis, not if it was part of empirical therapy. 

## 3. Results

### 3.1. Patients

In the studied period, four patients were referred to our ophthalmology department with fungal keratitis. All patients were female, wore soft contact lenses (for daily wear and monthly changed) and mean age was 46 years (range 27–67). None of the cases mentioned trauma to the eyes in their recent medical history, one of them had recently returned from a holiday abroad where she had been diving. The cultures grew *Fusarium* species in all patients. Below, we summarize the cases treated with CHX monotherapy. Further demographic and clinical details are depicted in Table 1.

**Figure 1:**
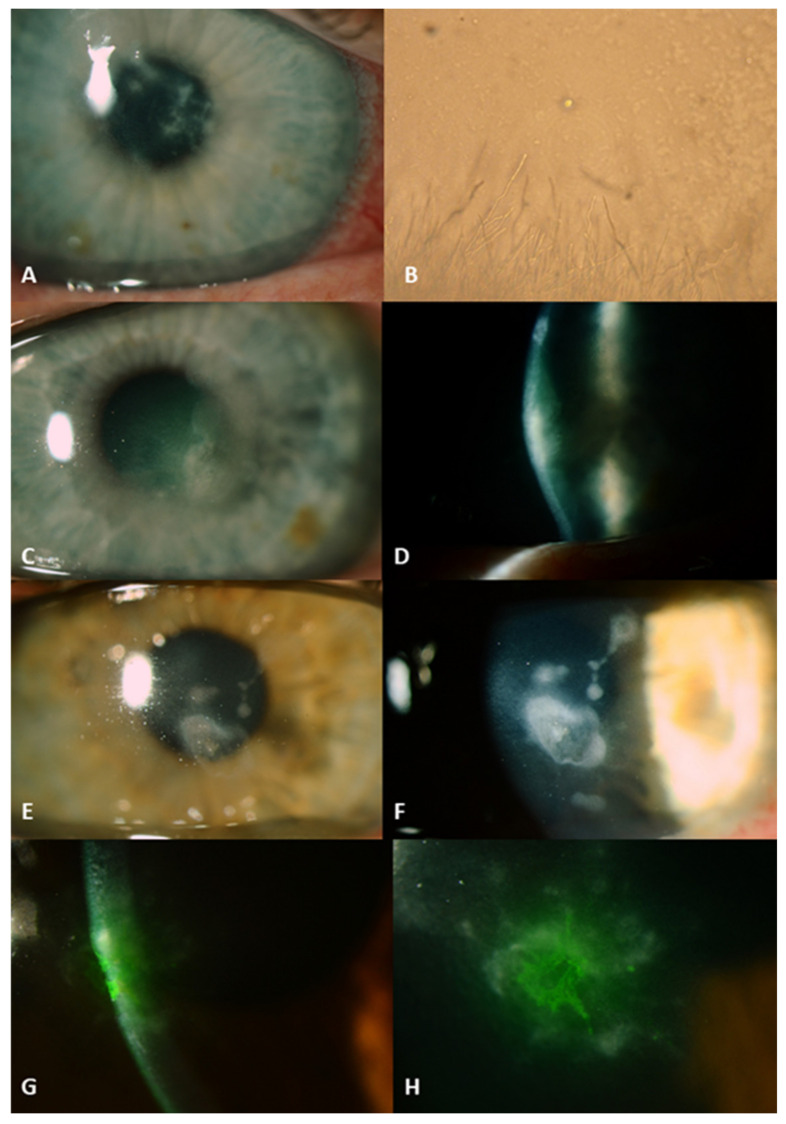
Fungal keratitis ulcers before the start of antifungal therapy with chlorhexidine digluconate. Patient from introduction: right eye with multiple infiltrates in the edematous corneal stroma (**A**). Culture of *Acanthamoeba* and *F. solani* on a nutrient agar with a lawn of *Enterobacter aerogenes*, magnification of 100× (**B**). Patient 1: left eye with infiltrates in the corneal stroma (**C**,**D**). Patient 2: left eye with infiltrates in the corneal stroma (**E**,**F**). Patient 3: cornea of the right eye stained with fluorescein showing infiltrates in corneal stroma (**G**,**H**).

All four patients presented with infiltrates with satellites in the superficial layers of the cornea, in less than 50% depth of the stroma (Figure 1). All were already treated with antibiotics (chloramphenicol or ofloxacin). One patient was on local steroids for three weeks. After taking specimens for microbiological analysis, we started empirical treatment with chlorhexidine monotherapy hourly for 48 h. After microbiological confirmation of the clinical diagnosis all patients continued the CHX as monotherapy, because a beneficial effect was already seen after 2 days. The prescribed treatment was continued for at least 6 weeks in a diminishing dose (minimal dose 4–6 dd). After resolution of the inflammation, all four eyes had visual acuity of 0.9 or more with a small paracentral scar.

In vitro chlorhexidine was active against the strains isolated from the cases. Furthermore, chlorhexidine showed fungicidal activity (MFC data not shown) within one twofold dilution step of the MIC in all strains, in other words MIC equals MFC. The susceptibility profiles of the fungal strains are summarized in Table 2.

### 3.2. Review

The search strategy yielded 38 hits, of which nine were possibly eligible based on the title and abstract. The availability of two of them were abstract only and were discarded because the articles did not meet the inclusion criteria (CHX not targeted for a fungal cause of the keratitis) [7,8]. Of the remaining seven publications, only three were eligible and included: two randomized controlled trials (RCTs) and one case report [9,10,11,12,13,14,15]. 

Rahman et al., performed both of the RCTs with CHX for fungal corneal ulcers. The first study was double blinded, included 60 cases of Indian patients and aimed to obtain the optimal CHX concentration for further studies. Therefore, they studied CHX in three different concentrations (0.005%, 0.1% and 0.2%) versus natamycin 5% (referent) [9]. CHX was found to be safe with regard to toxicity and to be superior to natamycin in all the used concentrations, of which CHX 0.2% was the most effective. The limitations of this study were the possible detection bias in blinding the outcome assessment and the possibility of selectivity in reporting different outcome measures.

In the second RCT of Rahman et al., CHX 0.2% versus natamycin 2.5% was studied in Bangladesh [10]. They included 70 participants in this trial. The results confirmed the superiority of CHX versus natamycin. However, there was risk of bias due to the fact that the medications had different appearances and due to differences in dropouts between the two groups (37.1% in CHX group versus 8.3% in the natamycin group). 

Ben-Simon, Barequet and Grinbaum described a patient with keratitis due to *Exophiala jeanselmei* after slight trauma to the eye [11]. She was successfully treated with a combination of natamycin 5%, amphotericin 5 mg/mL and chlorhexidine 0.02%. Even though this case was successfully treated, it is only one case where CHX was combined with two antifungal agents.

## 4. Discussion

Fungal keratitis is a serious infection that may have severe consequences and is generally difficult to treat. Currently, there are no clear guidelines for the management of fungal keratitis.

The successful treatment of our cases with chlorhexidine show that it seems to be a promising therapeutic agent in the management of fungal keratitis. Our susceptibility data suggest that the standard chlorhexidine concentration used for the treatment of *Acanthamoeba* keratitis (0.02%) may also be effective for the treatment of fungal keratitis. This is also supported by published in vitro data [16,17,18]. The main difficulties with topical antifungal treatment of ocular infections are poor ocular penetration and local bioavailability, the limited number of preparations, and drug toxicity. In both clinical trials and the case report, in the aforementioned literature review, chlorhexidine was well tolerated, and no clinically relevant toxicity was observed. 

Chlorhexidine belongs to the biguanides and is a widely used biocidal antisepticum with a broad spectrum of activity against bacteria, lipid enveloped viruses, *Acanthamoeba* species and fungi [19]. The mode of action of chlorhexidine in yeasts is similar to that in bacteria. After damage to the outer cell layers, CHX crosses the cell wall and impairs the integrity of the plasma membrane, resulting in leakage of cell contents and cell death. However, there is a biphasic effect on the permeability of the membranes at high concentrations [20,21]. This effect leads to coagulation of the cytosol resulting in reduced leakage. It is not known if this is a clinically relevant effect in fungal keratitis. There is little known about the ways in which fungi can circumvent the action of chlorhexidine, which could lead to resistance. 

Penetration of topical antifungal agents occurs mainly by diffusion through the cornea. Therefore, the molecular mass of the used agent is important to take into consideration. For example, the polyenes (amphotericin B and natamycin) barely penetrate the cornea due to their high molecular mass (>660 Da) [22]. Chlorhexidine has a lower molecular mass and penetrates the cornea better but still not completely; it appears to accumulate within the cornea [23]. Vontobel et al. showed (in a small animal study) that chlorhexidine did not penetrate through the intact or mechanically damaged cornea all the way into the anterior chamber [23]. 

From an epidemiological point of view, the cultured species in these cases and within the Netherlands *Fusarium* species are the most prevalent fungal species that cause fungal keratitis. This is in conjunction with the global species distribution described by Brown et al., who reported *Fusarium* as the most common causative agent followed by *Aspergillus* and *Candida* [24]. However, it is worth noting that there are regional differences to keep in mind.

We suggest the following treatment strategy regarding the use of chlorhexidine in fungal keratitis. In cases that are clinically suspect for fungal keratitis, we suggest starting chlorhexidine 0.02% hourly for 48 h. In cases with infiltrates as small as 1–2 mm and depth less than one third of the cornea, await the outcome of the culture, susceptibility testing and molecular testing if available. In larger and deeper infiltrates, additional application of voriconazole (1%) or amphotericin B (0.15%) is advocated. Voriconazole as monotherapy has been shown to be less effective than a polyene (natamycin or amphotericin B) but could have a beneficial additional effect in combination with CHX.

Overall, we can conclude that there is encouraging evidence for the use of chlorhexidine in the treatment of fungal keratitis. Advantages of chlorhexidine are topical application, general availability, very low costs, broad-spectrum activity, and fungicidal mechanism of action at relatively low concentrations. Further research is needed to investigate the extensiveness of the claimed broad-spectrum activity of CHX. This would make chlorhexidine a great candidate for empirical therapy of microbial keratitis, as well as for targeted treatment in fungal keratitis.

## Figures and Tables

**Table 1:** Demographic characteristics and outcome of fungal keratitis patients treated with chlorhexidine digluconate.

	Case 1	Case 2	Case 3	Case 4
Sex (M/F) and age (years)	F 67	F 27	F 36	F 52
Contact lens (cl) wear	Soft cl (monthly changed)	Soft cl (monthly changed)	Soft cl (monthly changed)	Soft cl (monthly changed)
Characteristics at presentation				
Visual acuity (Snellen)	0.05	0.5	0.6	1.0
Duration of complaints (days)	6	5	32	7
Infiltrate (mm/depth)	1 × 1/50%	1 × 0.4/<50%	1 × 1	1 × 1
Satellites present	Yes: multiple	Yes: multiple	Yes: multiple	No
Endothelium	Inflammatory cells	No	No	No
Inflammation in anterior chamber	No	No	No	1+
Pre-diagnosis: therapy				
Antibiotics (duration)	Ofloxacin	Gentamycin/cefazoline (2 days)	Ofloxacine (3 weeks)	Ofloxacine (1 week)
Antifungals	None	Amphotericine B/voriconazole (2 days)	None	None
Steroids	None	None	Yes: 3 weeks	None
Culture of cornea scraping				
	*Fusarium proliferatum* (*F. fujikuroi species complex*)	*Fusarium falciforme* (*F. solani species complex*)	*Fusarium verticillioides* (*F. fujikuri species complex*)	*Fusarium oxysporum* (*F. osysporum species complex*)
	*Cutibacterium acnes* *			Cutibacterium acnes *
Treatment after diagnosis				
Chlorhexidine 0.02% (max-min) **	24 dd >> 8 dd	12 dd >> 6 dd	24 dd >> 6 dd	24 dd >> 6 dd
Duration (days)	120	42	42	42
After resolution of fungal keratitis				
Days after targeted therapy	120	42	42	42
Visual acuity (Snellen)	0.9	1.0	1.0	1.0
Endothelial cel count	1980	NP ***	3380	NP ***

* Formerly known as *Propionibacterium acnes.* ** Topical treatment started with a maximum of 24 times per day (hourly droplets) and overtime was tapered to a minimum of 6 times per day. *** NP; not performed.

**Table 2:** In vitro activity of eight antifungal agents and chlorhexidine digluconate against the *Fusarium* strains of the described fungal keratitis cases.

	MIC % (mg/L)	MIC mg/L					MEC mg/L		
	CHX	AMB	ITC	VCZ	POS	ISA	ANI	CAS	MCF
*F. proliferatum* (*case 1*)	0.0008 (4)	0.5	>16	4	1	16	>16	>32	>2
*F. falciforme* (*case 2*)	0.0064 (32)	2	>16	>16	16	16	16	>32	>2
*F. verticillioides* (*case 3*)	0.0032 (16)	2	>16	2	0.5	16	16	>16	>2
*F. oxysporum* (*case 4*)	0.0004 (2)	1	ND	>16	>16	ND	ND	>32	ND

MIC, minimum inhibitory concentration; MEC, minimum effective concentration; CHX, chlorhexidine; AMB, amphotericin B; ITC, itraconazole; VCZ, voriconazole; POS, posaconazole; ISA, isavuconazole; ANI, anidulafungin; CAS, caspofungin; MCF, micafungin; ND: not determined.

## Data Availability

The data presented in this study are available on request from the corresponding author. The data are not publicly available due to e.g., privacy and ethics.
